# Repetitive Fed-Batch: A Promising Process Mode for Biomanufacturing With *E. coli*

**DOI:** 10.3389/fbioe.2020.573607

**Published:** 2020-11-10

**Authors:** Julian Kopp, Stefan Kittler, Christoph Slouka, Christoph Herwig, Oliver Spadiut, David J. Wurm

**Affiliations:** Research Area Biochemical Engineering, Institute of Chemical Engineering, TU Wien, Vienna, Austria

**Keywords:** *E. coli*, repetitive fed-batch, process understanding, process intensification, recombinant protein production, continuous biomanufacturing

## Abstract

Recombinant protein production with *Escherichia coli* is usually carried out in fed-batch mode in industry. As set-up and cleaning of equipment are time- and cost-intensive, it would be economically and environmentally favorable to reduce the number of these procedures. Switching from fed-batch to continuous biomanufacturing with microbials is not yet applied as these cultivations still suffer from time-dependent variations in productivity. Repetitive fed-batch process technology facilitates critical equipment usage, reduces the environmental fingerprint and potentially increases the overall space-time yield. Surprisingly, studies on repetitive fed-batch processes for recombinant protein production can be found for yeasts only. Knowledge on repetitive fed-batch cultivation technology for recombinant protein production in *E. coli* is not available until now. In this study, a mixed feed approach, enabling repetitive fed-batch technology for recombinant protein production in *E. coli*, was developed. Effects of the cultivation mode on the space-time yield for a single-cycle fed-batch, a two-cycle repetitive fed-batch, a three-cycle repetitive fed batch and a chemostat cultivation were investigated. For that purpose, we used two different *E. coli* strains, expressing a model protein in the cytoplasm or in the periplasm, respectively. Our results demonstrate that a repetitive fed-batch for *E. coli* leads to a higher space-time yield compared to a single-cycle fed-batch and can potentially outperform continuous biomanufacturing. For the first time, we were able to show that repetitive fed-batch technology is highly suitable for recombinant protein production in *E. coli* using our mixed feeding approach, as it potentially (i) improves product throughput by using critical equipment to its full capacity and (ii) allows implementation of a more economic process by reducing cleaning and set-up times.

## Introduction

*Escherichia coli* serves as a beloved workhorse for the production of many recombinant proteins. Fast doubling times, little risk of contamination, cheap media and easy upscale are the most prominent benefits (Casali, 2003; Baeshen et al., 2015; Gupta and Shukla, 2017). The *E. coli* strain BL21(DE3) is the most commonly applied strain in industry with outstanding low acetate secretion and high product concentrations (Rosano and Ceccarelli, 2014; Wurm et al., 2017a; Rosano et al., 2019). The strain is regularly used for recombinant protein production with pET-plasmids, making use of the integrated T7-promotor system (Wurm et al., 2016; Kopp et al., 2017; Rosano et al., 2019). For many applications IPTG (Isopropyl-β-D-thiogalactopyranosid) is the inducer of choice, which leads to high levels of recombinant protein. Even though IPTG induction is described as tunable, toxic effects can be observed (Wurm et al., 2016; Hausjell et al., 2018). Several studies showed that the use of IPTG throughout long induction times leads to increased stress levels and thus to low viability (Dvorak et al., 2015; Slouka et al., 2018). Promotor systems, like araBAD and rhamBAD are not described to show any toxic effects (Marschall et al., 2016). The utilization of arabinose or rhamnose might enable long-term cultivation with *E. coli* (Marschall et al., 2016), however they are rarely used in industry due to the high price of these inducers (Kopp et al., 2019b). Thus, it is of great importance to find a suitable inducer that is affordable in large scale and shows no signs of toxicity onto host cells (Malakar and Venkatesh, 2012; Mühlmann et al., 2018). The disaccharide lactose is taken up via lactose permease. Upon uptake, lactose is either cleaved to glucose and galactose or converted to allolactose via β-galactosidase (Deutscher et al., 2006). Allolactose can then bind to the lac repressor and enable induction as described in various previous publications (Wurm et al., 2016; Kopp et al 2017). Due to the non-toxicity and low cost of lactose compared to other inducers, this induction mechanism is tuneable and also economically feasible (9.39 €/g IPTG vs. 0.02 €/g lactose) (Yan et al., 2004; Briand et al., 2016). For mixed feed systems using lactose, an established mechanistic knowledge platform, which explains the correlation between sugar and inducer uptake by physiological parameters can be used (Wurm et al., 2017a). Furthermore, lactose has shown to boost productivity in soluble recombinant protein production when compared to IPTG (Wurm et al., 2016, 2017a). For periplasmic recombinant protein production soft induction by lactose is especially important as translocation to the periplasm is the rate limiting step (Gupta and Shukla, 2017; Karyolaimos et al., 2019; Hausjell et al., 2020).

Independent of product location, recombinant protein production in *E. coli* is commonly carried out in fed-batch cultivation mode (Slouka et al., 2018; Kopp et al., 2019b). However, in fed-batch cultivation sterilization, cleaning and biomass formation take up the majority of process time (Slouka et al., 2018). As industry is always aiming to increase the space-time yield, a continuous production system would be desirable to reduce down-times (Rathore, 2015; Tan et al., 2019; Zobel-Roos et al., 2019). Regulations of continuously produced products used to be an issue, however regulatory authorities have defined to separate production into diverse lot numbers according to ICH Q7: “The batch size can be defined either by a fixed quantity or by the amount produced in a fixed time interval” (EU GMP Guide, Part II). Compared to a batch system, continuous systems enable maximum utilization of equipment. By reducing down-times, production scale can be decreased or amounts of product can be gained within less time, or a combination of both factors (Glaser, 2015; Herwig et al., 2015; Rathore, 2015). Continuous production processes would allow increased product yields in smaller production facilities while obtaining the same amounts of product (Allison et al., 2015; Konstantinov and Cooney, 2015; Nasr et al., 2017).

Aiming to establish time-independent microbial cultivation systems, evolutionary mechanisms, such as mutations (Rugbjerg and Sommer, 2019) and shifts in transcriptome and proteome (Peebo et al., 2015; Peebo and Neubauer, 2018) spoiled expectations of industry.

Repetitive fed-batch cultivation mode offers the chance of an immense down-time reduction, with multiple production cycles performed within one cultivation run (Bergmann and Trösch, 2016; Kuo et al., 2017; Zagrodnik and Łaniecki, 2017). While in fed-batch processes a complete harvest of the fermenter is performed at the end of cultivation, repetitive fed batch processing differs by performing only a partial harvest (Martens et al., 2011). Afterwards, the spared fermentation broth is diluted with fresh media and a new induction cycle can be started right away (Fricke et al., 2013). Repetitive fed-batch has proven to be a suitable cultivation mode to improve many processes in biotechnology and conducted studies and literature concerning repetitive fed-batch cultivations up to date are given in Table 1. However, studies on repetitive fed-batch using *E. coli* are scarce.

**TABLE 1:** Summary of studies about repetitive fed-batch cultivations.

Microorganism	Product	Process description	Ref.
*Crypthecodinium cohnii*	docosahexaenoic acid	4 cycles, 80% medium replacement	Liu et al., 2020
*Aspergillus terreus*	Lovastatin	3 cycles, 37% yield in crease	Novak et al., 1997; Kumar et al., 2000
*Gluconobacter oxydans*	Dihydroxyacetone	4 cycles, repeated fed-batch process using two spatially separated vessels	Bauer et al., 2005
*Kluyveromyces marxianus*	Ethanol	5 cycles, product yield constant	Ozmihci and Kargi, 2007
*Kitasatospora*	ε-Poly-L-lysine	5 cycles	Zhang et al., 2010
*Yarrowia lipolytica*	Citric acid	10 cycles, productivity decrease over cultivation time	Moeller et al., 2011
*Pichia pastoris*	human serum albumin (rHSA)	4 cycles, 47% yield increase	Ohya et al., 2005
*Pichia pastoris*	Malaria vaccine candidates	stable productivity for 2-8 cycles, methanol induction	Martens et al., 2011; Fricke et al., 2013
*Escherichia coli*	Pyruvate	5 cycles, q_p_ increased fivefold	Zelić et al., 2004

Repetitive fed-batch technology has shown promising effects in recombinant protein production mainly using *Pichia pastoris* (Ohya et al., 2005; Martens et al., 2011; Fricke et al., 2013). To our knowledge, a repetitive fed-batch cultivation mode using *E. coli* has only been implemented for pyruvate production (Zelić et al., 2004). However, the potential of using *E. coli* in repetitive fed-batch mode for recombinant protein production has not been investigated yet.

In this study we performed repetitive fed-batch cultivations for recombinant protein production using the production host *E. coli* in combination with lactose induction. In previous studies, the negative side effects of IPTG induction onto recombinant protein production in long-term fermentations were shown, whereas lactose was found to have a beneficial effect on productivity (Malakar and Venkatesh, 2012; Dvorak et al., 2015; Kopp et al., 2019a). We believe that no studies on repetitive fed-batch cultivation with *E. coli* for recombinant protein production have been published yet, either due to toxic effects of IPTG and the consequent decreasing productivity over time (Dvorak et al., 2015; Kopp et al., 2019b) or due to the absence of an induction strategy comparable to the established yeast system (Fricke et al., 2013). The goal of this study was to compare productivities and space-time yields of different production modes for *E. coli.* The assessment of changes in product quality and purity was not in the scope of this study. For the first time, we were able to show that a repetitive fed-batch cultivation mode using our developed lactose induction strategy is able to outperform conventional fed-batches and chemostat cultivations regarding the overall space-time yield.

## Materials and Methods

### Strains

All cultivations were carried out with the strain *E. coli* BL21(DE3), transformed with a pET30a^+^ plasmid carrying the gene for the cytoplasmic protein (CP) and periplasmic protein (PP), respectively. The cytoplasmic protein contained no disulfide bonds, had a isoelectric point (PI) of 5.62 and a protein size of 26.9 kDa. PI and protein size of the periplasmic protein were 5.42 and 32 kDa, respectively and it contained a single disulfide bond.

### Media

All cultivations were conducted using a defined minimal medium by DeLisa et al. (1999), supplemented with different amounts of glucose and lactose. 0.02 g/L kanamycin was added to prevent plasmid loss.

### Bioreactor Setup

All cultivations were performed in a Minifors 2 bioreactor system (max. working volume: 1 L; Infors HT, Bottmingen, Switzerland). The cultivation offgas was analyzed in online mode using gas sensors – IR for CO_2_ and ZrO_2_ based for Oxygen (Blue Sens Gas analytics, Herten, Germany).

Process control and exponential feeding was established using the process control system PIMS Lucullus (Securecell, Urdorf Switzerland). pH was monitored using an EasyFerm Plus pH-sensor (Hamilton, Reno, NV, United States) and was kept constant at 6.7 throughout all cultivations and controlled using a base only control (12.5% NH_4_OH), while acid (5% H_3_PO_4_) was added manually, if necessary. Stirrer speed was set to 1400 rpm. Dissolved oxygen (dO_2_) was kept above 30% oxygen saturation by supplying 2 vvm of a mixture of pressurized air and pure oxygen. The dO_2_ was monitored using a Visiferm fluorescence dissolved oxygen electrode (Hamilton, Reno, NV, United States). Feed medium was added by using a PRECIFLOW pump (Lambda, Laboratory Instruments, Baar, Switzerland). Reactor weight and the depleted feed weight were monitored to determine exact feeding rates using a feed forward control as described here (Slouka et al., 2018). Harvest and fill-up step were conducted using a peristaltic pump (Watson-Marlow, Guntramsdorf, Austria).

### Cultivation Procedure

The pre-culture and batch phase was equivalent for all performed cultivations, followed by a single-cycle fed-batch, a two-cycle fed-batch, a three-cycle fed-batch or a chemostat cultivation (Supplementary Figure 1).

#### Pre-culture

Pre-culture was prepared using 2500 mL high yield flasks. 500 milliliter of DeLisa medium (DeLisa et al., 1999) supplemented with 8.8 g/L glucose were inoculated with 1.5 mL of bacteria solution stored in cryos at −80°C and subsequently cultivated for 16 h at 230 rpm in an Infors HR Multitron shaker (Infors, Bottmingen Switzerland) at 37°C.

#### Batch Cultivation

Batch medium [DeLisa medium supplement with 20 g/L glucose (DeLisa et al., 1999)] was inoculated with 1/10th of the reactor volume using the previously described pre-culture. Batch process was carried out at 37°C and took approximately 6–7 h until sugar was depleted, monitored via a drop in the CO_2_ signal or a pO_2_ peak, respectively.

### Fed-Batch for Biomass Generation

After the batch phase, a non-induced fed-batch was carried out at 35°C over-night using carbon-limited feeding approaches. Non-induced fed-batch was carried out at a constant specific feeding rate (=q_s_) of 0.25 g/g/h to achieve a biomass concentration of approximately 35 g/L prior to induction (Supplementary Figures 3a,b). For exponential feeding, DeLisa medium (DeLisa et al., 1999) supplemented with 300 g/L glucose was used as feed medium. Equation 1 was used for the feed controller to calculate the feed-rate for maintaining a constant q_s_ in feed forward mode (Kopp et al., 2017; Wurm et al., 2017a). Dry cell weight of biomass and feeding rates were determined as described here (Hausjell et al., 2018; Slouka et al., 2018). In short, triplicate at-line optical density (OD) measurements and a previously established OD to biomass correlation were used for calculation of the exponential feeding profile.

(1) $$F\left(t\right)=\frac{q_{s}\times X\left(t\right)\times \rho_{f}}{c_{f}}.$$

F, feed-rate (g/h); q_s_, specific glucose uptake rate (g/g/h); X(t), biomass (g); ρ_f_, feed density (g/L); c_f_, feed concentration (g/L).

#### Single-Cycle Fed-Batch

**FIGURE 1:**
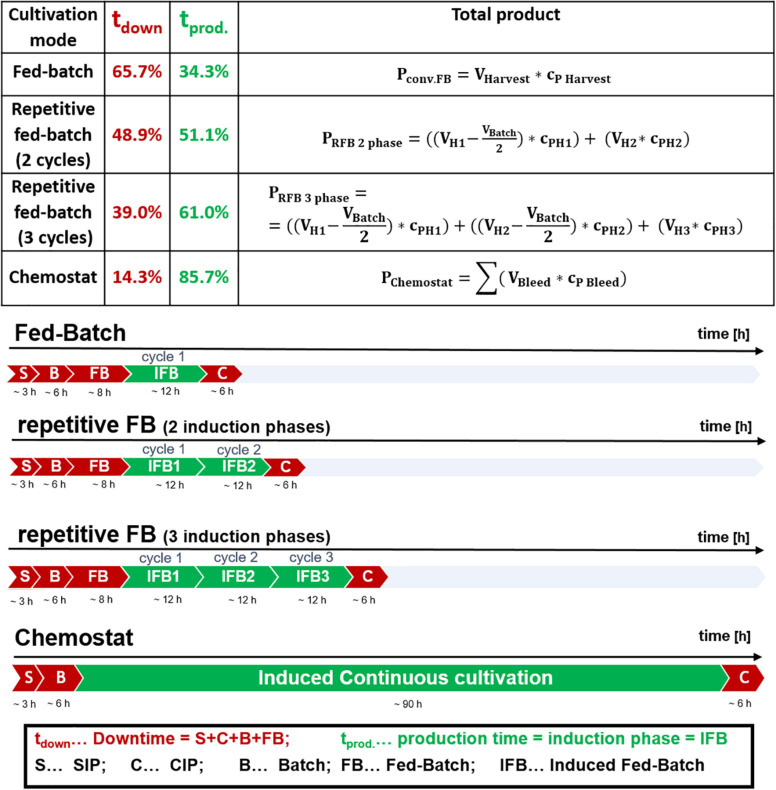
Comparison of cultivation durations of a fed-batch, a repetitive fed-batch consisting of two cycles, a repetitive fed-batch consisting of three cycles and a chemostat process; effective production time vs. downtime for a 10 m^3^ fermenter scale is given for each process in percent relative to total process time; steam in place (SIP), cleaning in place (CIP).

Prior to induction, temperature was decreased to 30°C, to reduce stress onto host cells and enhance soluble protein formation (Wurm et al., 2016). The induction phase was conducted for 12 h using a glucose–lactose mixed feed system according to a previous study (Slouka et al., 2018). For induction, DeLisa medium (DeLisa et al., 1999) supplemented with 250 g/L glucose and 141.2 g/L lactose, was fed at a constant specific feeding rate of 0.25 g/g/h, as these mixing ratios were found to show better results in previous cultivations (Wurm et al., 2017a). For single-cycle fed-batch cultivations, the cultivation was terminated after 12 h of induction and a full reactor harvest was conducted (Figure 1 and Supplementary Figure 1a), whereas for two-cycle and three-cycle fed-batches only a partial harvest was performed.

#### Two-Cycle Repetitive Fed-Batch

Prior to the repetitive fed-batch the process cycles preculture, batch cultivation, fed-batch for biomass generation and the single-cycle fed-batch were performed. However, after the induction phase feeding was stopped and only a partial harvest was conducted leaving half of the initial volume for the ongoing process steps. In order to achieve the same biomass concentration as before induction, biomass was determined at-line and diluted to approximately 35 g/L using sterile DeLisa medium (DeLisa et al., 1999). Dilution media contained no carbon source but was double concentrated in salt and trace element concentration to avoid nutrient limitation throughout the following repeated cycles. Antibiotic was also added to the sterile medium to achieve the initial start concentration. After the refilling the feeding was started analogous to the single-cycle fed-batch for another 12 h. Complete harvest was conducted after the second cycle.

#### Three-Cycle Repetitive Fed-Batch

Cultivation was carried out analog to the description in section “Two-Cycle Repetitive Fed-Batch.” However, after the second cycle was finished, again only a partial harvest was conducted. The refilling step was conducted analog to the procedure for the two-cycle repetitive fed-batch and the total fermentation broth was harvested after cycle 3 was finished.

#### Chemostat Cultivation

After batch cultivation the continuous process mode was started. Dilution rate was set to D = 0.1 h^–1^ and the volume in the reactor was kept constant at 750 mL using an immersion tube adjusted to the right height of the stirred liquid surface in the reactor which was connected to a bleed pump (Watson-Marlow, Guntramsdorf, Austria). Medium for chemostat cultivation was prepared as described by DeLisa (DeLisa et al., 1999) supplemented with 50 g/L glucose and 25 g/L lactose. To keep the growth rates of the performed repetitive fed-batch processes comparable to the performed chemostat processes, only dilution rates of 0.1 h^–1^ were investigated within this study. The overall induction time of the chemostat process was 90 h.

#### Ideal Chemostat

**FIGURE 2:**
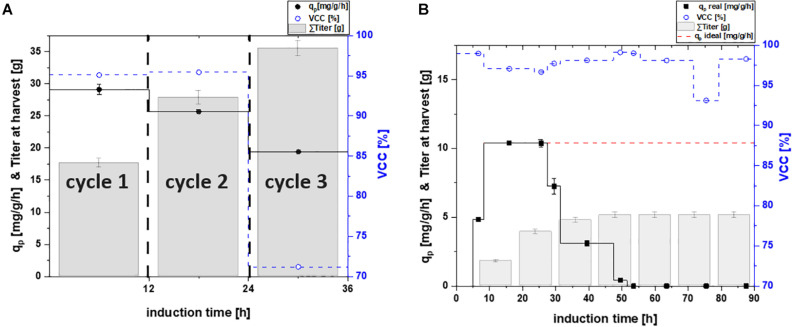
Specific productivity q_p_ (mg/g/h), viable cell concentration (=VCC) [%] and the harvested product titer for **(A)** a repetitive fed-batch cultivation and **(B)** a chemostat process; a theoretical ideal chemostat was simulated at q_p, max_.; VCC was evaluated by flow cytometry analysis with an average standard deviation of 2%.

**FIGURE 3:**
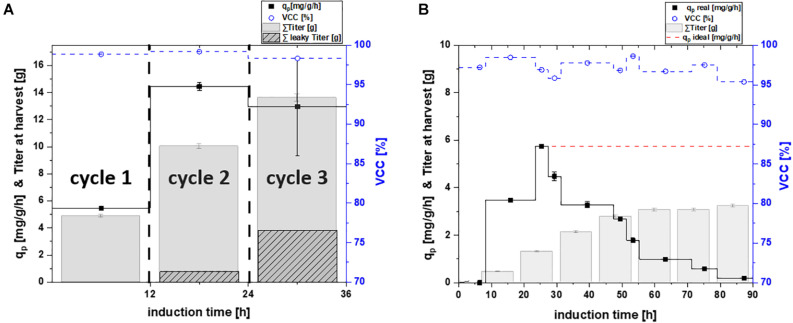
Specific productivity q_p_ (mg/g/h), viable cell concentration (=VCC) [%] and the titer available for harvest plotted for **(A)** repetitive fed-batch cultivation separated in their number of cycles; and **(B)** a chemostat process plotted as a function of induction time; theoretical ideal chemostat was simulated at q_p, max_.; as extracellular protein was found for cultivations of the periplasmic proteins, the secreted protein is shown in dotted lines for the harvested titer; VCC was evaluated by flow cytometry analysis with an average standard deviation of 2%.

In order to test advantages of a continuous cultivation system, an ideal chemostat was simulated. We calculated the ideal chemostat with stable product formation at maximum specific productivity as shown in Figures 2B,3B.

### Sampling

Samples were taken at the end of the batch phase, after the fed-batch phase and regularly during the induced cycles of all cultivation modes. Biomass, optical density, viability and metabolite accumulation were determined for every sample taken. All samples taken during induction phase were additionally analyzed for product formation.

Samples during repetitive fed-batch cultivations were taken every three hours during the first and the third cycle, while the second cycle was conducted over night and therefore only the harvest sample was taken.

For the chemostat cultivation, samples were taken 3 h after start of the induced chemostat and from then on twice a day.

#### Biomass, Viability, Substrate and Metabolite Analysis

Biomass was measured by optical density (OD_600_) and gravimetrically by dry cell weight (DCW), while flow cytometry analysis (FCM) was used for the determination of viability.

OD_600_ was measured in triplicates using a Genesys 20 photometer (Thermo Scientific, Waltham, MA, United States). Since the linear range of the used photometer was between 0.2 and 0.8 (AU), samples were diluted with dH_2_O to stay within the given range.

The DCW was determined by centrifugation (10,000 rpm for 10 min at 4°C) of 1 mL of homogenous sample solution in a pre-tared 2 mL Eppendorf-Safe-Lock Tube (Eppendorf, Hamburg, Germany). After centrifugation, the supernatant was withdrawn, frozen at −20°C and used to determine sugar concentrations by HPLC measurements. The pellet was re-suspended with 1 mL of 0.9% NaCl solution and centrifuged again (10,000 rpm for 10 min at 4°C). Afterward, the pellet was dried for at least 48 h at 105°C and DCW was evaluated gravimetrically in triplicates.

For FCM, cultivation broth was diluted 1:100 with 0.9% NaCl solution, stored at 4°C and measured every day. The measurement was performed using a Cyflow^®^ Cube 8 flow cytometer (Sysmex, Görlitz, Germany) according to Langemann et al. (2016) using DiBAC4(3) (bis-(1,3-dibutylbarbituricacid-trimethineoxonol) and Rh414 dye. Both dyes were purchased from AnaSpec (Fremont, CA, United States).

Sugar concentrations of feed and clarified fermentation broth were measured via anion exchange HPLC (Thermo Scientific, Waltham, MA, United States) using a Supelcogel-column (Sigma-Aldrich, St. Louis, MO, United States) and a refractive index detector (Agilent Technologies, Santa Clara, CA, United States). The mobile phase was 0.1% H_3_PO_4_ with a constant flow rate of 0.5 mL/min, and the system was run isocratically at 30°C. Glucose, lactose, galactose, and acetate accumulation was monitored using calibration standards with a concentration of 1, 5, 10, 25, and 50 g/L of each analyte. Chromatograms were analyzed using Chromeleon Software (Thermo Scientific, Waltham, MA, United States).

#### Product Quantification

Product samples were taken after the start of the induction phase. Five milliliter of cultivation broth were pipetted in a 50 mL falcon tube and centrifuged for 10 min at 5000 rpm at 4°C. The supernatant was discarded and the pellet was frozen at −20°C. Afterward, the samples were disrupted by homogenization as follows: The pellets were re-suspended in a buffer (0.1 M TRIS, 10 mM EDTA, pH 7.4) according to their dry cell weight to reach a biomass of 10 g/L prior to homogenization. After suspending the cells with a disperser (T10 basic ULTRA-TURRAX^®^, Staufen, Germany) they were treated with an EmusiflexC3 Homogenizer (Avestin, Ottawa, ON, United States) at 1400 bar for 4 passages, ensuring complete cell disruption. After homogenization the broth was centrifuged (14,000 rpm, 10 min, 4°C) and the supernatant was used immediately for HPLC quantification.

For soluble titer measurements of the cytoplasmic target protein, the supernatant derived after centrifugation of homogenized broth was filtered and then quantified via UHPLC (Thermo Scientific, Waltham, MA, USA). For quantification of cytoplasmic soluble protein, a size exclusion (=SEC) chromatography principle was applied, using a X-bridge column (Waters, Milford, DE, United States). The mobile phase was composed of 250 mM KCl and 50 mM of each KH_2_PO_4_ and K_2_HPO_4_ dissolved in Ultrapure water as describe elsewhere (Goyon et al., 2018). A constant flow rate of 0.5 mL/min was applied with an isocratic elution at 25°C for 18 min. BSA standards (50, 140, 225, 320, 500, 1000, and 2000 mg/mL; Sigma Aldrich, St. Louis, MO, United States) were used for quantification.

For soluble titer measurements of the periplasmic protein, clarified cultivation broth was analyzed by a BioResolve RP mAb Polyphenyl column (Waters, Milford, DE, United States), using a reversed-phase HPLC method published elsewhere (Kopp et al., 2020). Product was quantified with a UV detector (Thermo Fisher, Waltham, MA, United States) at 214 nm, using BSA as standard reference.

Specific productivity was calculated as a rate between two sampling points using Eq. 2:

(2) $$q_{p}=\frac{\frac{c_{i}+c_{i-1}}{2}}{\frac{x_{i}+x_{i-1}}{2}}\times \frac{1}{t_{i}-t_{i-1}}.$$

*q*_p_ specific productivity (mg/g/h); *c*_*i*_, product concentration of sample at timepoint *i* (mg/L); *X*_*i*_, biomass concentration of sample at timepoint *i* (g/L); *t*_*i*_, cultivation time at timepoint of sample *i* (h).

The experimentally evaluated q_p_ values were used for the calculation of the real chemostat. For the simulated ideal chemostat, stable product formation at maximum specific productivity was assumed, once the maximum productivity was reached, as shown in Figures 2B,3B.

### Reproducibility

To test the reproducibility of the equipment described in section “Bioreactor Setup,” triplicates of a fed-batch cultivation were assessed by the same operator for one target protein. Found errors were not higher than ±0.35 g/L for titer determination [resulting in a maximum relative standard deviation (RSD) below 10%]. Specific feeding rates were found to be within a deviation of ± 0.03 g/g/h (max. RSD below 11%). Dry cell weights deviations between replicates were below 3.8 g/L (below a max. RSD of 9%). Set specific feeding rates require at-line OD_600_ determination to estimate the biomass, before the exponential feeding ramp is calculated. Variances (i.e., due to dilution) in OD_600_-signals thus may cause differences in the resulting biomass and titer, as previously shown (Slouka et al., 2018).

## Results and Discussion

### Experimental Design

The potential of achieving high recombinant protein titers in repetitive fed-batch cultivation mode was shown by Luttmann et al. for *P. pastoris* as production host (Martens et al., 2011). However, repetitive fed-batch technology for recombinant protein production using *E. coli* has not been investigated yet. As methanol was continuously fed throughout the repetitive fed-batch studies with *P. pastoris* (Martens et al., 2011; Fricke et al., 2013), we established a similar feeding strategy for the inducer lactose and *E. coli*. Hence a feed-forward feeding strategy according to Eq. 1 was applied throughout all cycles. Furthermore, we tested whether a single-cycle fed batch, a two-cycle repetitive fed-batch, a three-cycle repetitive fed-batch or a chemostat is the cultivation mode of choice regarding overall space-time yield. We tested the cultivation modes for two different recombinant products: one produced in the cytoplasm and one secreted to the periplasm. Establishing such a process is of high interest for industry, to reduce downtime. For the calculations within this study the duration of the cycles and downtimes of the cultivation were chosen as regularly applied in industry (Slouka et al., 2018). Downtime, production time as well as calculation of total product titer are depicted in Figure 1.

The fermenter scale for the calculations was assumed with 10 m^3^, which is a common scale for *E. coli* in industry. Thus, time for steam in place (SIP) and cleaning in place (CIP) takes 3 and 6 h, respectively (communication with industrial partner). Batch phase on glucose was 6 h (Slouka et al., 2018). Non-induced fed-batch time was 8 h, to achieve a biomass of 35–40 g/L before induction using a growth rate of μ = 0.1 h^–1^ (equivalent to a q_s_ of 0.25 g/g/h using a biomass/substrate yield of 0.4 g/g for calculation, Kopp et al., 2017). Previous results indicate, that a q_s_ of 0.25 g/g/h, a cultivation temperature of 30°C and an induction time of 10–12 h is beneficial for the production of many recombinant proteins and was thus chosen for this study (Wurm et al., 2016; Wurm et al., 2017b; Slouka et al., 2018; Schein, 2019). As fed-batch cultivations were conducted at q_s_ = 0.25 g/g/h (equivalent to μ = 0.1 h^–1^), dilution rates in chemostat cultivation were investigated for the same μ = D = 0.1 h^–1^.

The final product titer for the different cultivations modes was calculated according to Figure 1. Space-time yield was calculated according to Equation 3 to allow comparison of the different process modes.

(3) $$STY=\frac{\sum V_{Harvest}\times c_{Protein}\times 24}{V_{Reactor}\times t}.$$

STY, space-time yield (g/L/day); Σ V_Harvest_, sum of harvested volume (L); c_Protein_, protein titer measured by HPLC analysis (g/L); V_Reactor_, Reactor volume (L); t, process time (h).

### Cultivation Strategies and Their Results for Cytoplasmic Protein Formation

Mixed feed approaches, containing glucose and lactose, were found to enhance soluble protein formation (Wurm et al., 2016, 2017a,b). This is in accordance with our results obtained for the single-cycle fed-batch cultivation for the cytoplasmic protein, yielding a specific productivity of 29.12 mg/g/h. Other studies indicate stable viability throughout fed-batch cultivation using lactose induction at given feeding rates (Kopp et al., 2017), which we also confirmed in this study (95.1% viability at harvest).

Throughout the two-cycle repetitive fed-batch cultivation a minor decrease in productivity from 29.12 to 25.67 mg/g/h could be observed. Even though productivity in cycle two decreased, the total titer obtained per liter reactor volume at harvest increased majorly (Table 2). As viability was also high with 95.4% throughout the second cycle, the reduction in cell specific productivity might be a result of metabolic burden. Kanamycin concentration was adapted to the starting concentration of 0.02 g/L before the start of each repeated cycle to avoid possible plasmid loss.

**TABLE 2:** Comparing specific productivity and the product titer at harvest for the production of a cytoplasmic protein; each cycle is investigated separately for fed-batch and repeated fed-batch cultivation; values for chemostat and theoretical “ideal” chemostat cultivations are given every 12 h (i.e., one cycle); calculation for q_p_ represents instantaneous values whereas titers are calculated as accumulated values.

induction time (h)	12		24		36		48		60		72		84	
Cytoplasmic protein	q_p_ (mg/g/h)	Titer (g/L)	q_p_ (mg/g/h)	Titer (g/L)	q_p_ (mg/g/h)	Titer (g/L)	q_p_ (mg/g/h)	Titer (g/L)	q_p_ (mg/g/h)	Titer (g/L)	q_p_ (mg/g/h)	Titer (g/L)	q_p_ (mg/g/h)	Titer (g/L)
Fed-batch (1 cycle)	29.12 ± 0.82	17.64 ± 0.67												
Repeated fed-batch (2 cycles)			25.66 ± 0.32	27.85 ± 1.05										
Repeated fed-batch (3 cycles)					19.42 ± 0.14	35.51 ± 1.16								
Chemostat culitvation	8.00 ± 0.13	1.80 ± 0.07	9.18 ± 0.15	3.92 ± 0.15	4.75 ± 0.08	4.77 ± 0.19	0.25 ± 0	5.14 ± 0.20	0.00	5.14 ± 0.2	0.00	5.14 ± 0.2	0.00	5.14 ± 0.2
Theroretical “ideal” chemostat	8.00 ± 0.13	1.80 ± 0.07	9.18 ± 0.15	3.92 ± 0.15	10.4 ± 0.17	6.84 ± 0.27	10.4 ± 0.17	9.18 ± 0.36	10.4 ± 0.17	11.52 ± 0.45	10.4 ± 0.17	13.86 ± 0.54	10.4 ± 0.17	16.2 ± 0.63

Throughout the third cycle, a rapid decrease of q_p_ to 19.51 mg/g/h was found. Viable cell concentration decreased by 30%, which is most likely the cause for the high decrease in productivity. Upon producing high amounts of recombinant protein the host cell needs to be divided into a physiological compartment and a recombinant compartment as described by Neubauer et al. (2003). High titers as needed to make recombinant protein formation industrially feasible can also be toxic for host cells. As accumulated recombinant protein can effect ATP and NADH balances negatively this might lead to a decrease in physiological functions of the cell and can potentially hinder cell doubling (Rugbjerg and Sommer, 2019). Even though the specific productivity declined over time, the harvested titers of cycle three (Figure 2A) still increased significantly, compared to the harvest of the previous cycles. As viability decreased majorly throughout the third cycle, no further cycle was conducted.

For the chemostat cultivation, product formation started after an adaption phase of 5 h post induction (Figure 2B). Specific productivity further increased until it peaked after 18 h of induction at a q_p_ of 10.4 mg/g/h. However, ongoing sampling points determined the product formation to decrease rapidly and to terminate after 50 h of induction. No decrease in viability was observed and kanamycin was continuously fed to the system to avoid plasmid loss. Still, reduced plasmid copy numbers might occur and thus could be an explanation for the decreasing productivity (Sieben et al., 2016). Recent results, however, show that the productivity can fluctuate in lactose induced chemostat with BL21(DE3) as a result of genotypic or phenotypic diversification (Kittler et al., 2020). Hence we believe effects causing the decrease in productivity derive from subpopulation diversification. Shifts in the transcriptome in combination with point mutations (Rugbjerg and Sommer, 2019), are known to cause the formation of non-producing subpopulations (Basan, 2018). Recent publications (Schreiber et al., 2016; Binder et al., 2017) showed that carbon limited feeding increases probability of phenotypic subpopulation diversification. These effects are described to increase with generation time (Rugbjerg et al., 2018). As cells in chemostat processes are cultivated for longer time-spans than fed-batch and repeated fed-batch processes, long-term cultures face a higher chance of being affected (Buerger et al., 2019). We believe that a fitter subpopulation, having altered levels of transcription, is avoiding the burden of production. As recombinant protein expression is referred to cause decreasing biomass yields, a non-productive subpopulation, showing no decrease in biomass yield thus could overgrow the initial population. Hence we believe that the productive subpopulation is washed-out over the time-course of the induction phase and a non-productive subpopulation takes over, explaining the decline in productivity (Peebo and Neubauer, 2018; Kopp et al., 2019b).

In order to test the applicability of continuous cultivations for industry, a theoretical “ideal” chemostat with constant productivity at maximum specific productivity was simulated. However, the maximum specific productivity during the chemostat cultivation is significantly lower compared to the repetitive fed-batch cultivation (2.9 times lower compared to productivity of cycle two, Figures 2A,B). Furthermore, higher biomass concentrations and thus higher volumetric titers can be achieved in fed-batch and repetitive fed-batch mode. As biomass yield is decreasing upon production of recombinant proteins, this can potentially lead to washout (Lis et al., 2019). Hence, trying to achieve “fed-batch like” biomass concentrations in chemostat cultivation is highly difficult. Our results are in favor of fed-batch and repetitive fed-batch cultivation for the cytoplasmic target protein and will be compared in section “Targeting Maximum Space-Time Yield: The Cultivation Mode to Choose” regarding their space-time yield.

### Cultivation Strategies and Their Results for Periplasmic Protein Formation

In order to test and verify the effects monitored for the cytoplasmic protein, we investigated the same cultivation techniques for periplasmic protein production (Figure 3 and Table 3).

**TABLE 3:** Comparing specific productivity and the product titer at harvest for the production of a periplasmic protein; each cycle is investigated separately for fed-batch and repeated fed-batch cultivation; values for chemostat and theoretical “ideal” chemostat cultivations are given every 12 h (i.e., one cycle); calculation for q_p_ represents instantaneous values whereas titers are calculated as accumulated values.

induction time (h)	12		24		36		48		60		72		84	
periplasmic protein	q_p_ (mg/g/h)	Titer (g/L)	q_p_ (mg/g/h)	Titer (g/L)	q_p_ (mg/g/h)	Titer (g/L)	q_p_ (mg/g/h)	Titer (g/L)	q_p_ (mg/g/h)	Titer (g/L)	q_p_ (mg/g/h)	Titer (g/L)	q_p_ (mg/g/h)	Titer (g/L)
Fed-batch (1 cycle)	5.47 ± 0.06	4.87 ± 0.09												
Repeated fed-batch (2 cycles)			14.44 ± 0.30	10.03 ± 0.19										
Repeated fed-batch (3 cycles)					12.96 ± 3.65	13.61 ± 0.26								
Chemostat culitvation	1.98 ± 0.03	0.45 ± 0.01	5.26 ± 0.08	1.30 ± 0.02	3.76 ± 0.06	2.12 ± 0.04	2.32 ± 0.04	2.78 ± 0.05	1.31 ± 0.02	3.06 ± 0.06	0.69 ± 0.01	3.07 ± 0.06	0.34 ± 0.01	3.22 ± 0.06
Theroretical “ideal” chemostat	1.98 ± 0.03	0.45 ± 0.01	5.26 ± 0.08	1.30 ± 0.02	5.75 ± 0.04	3.16 ± 0.05	5.75 ± 0.04	5.02 ± 0.07	5.75 ± 0.04	6.88 ± 0.10	5.75 ± 0.04	8.74 ± 0.12	5.75 ± 0.04	10.6 ± 0.14

The measured cell specific productivity in the single-cycle fed-batch was q_p_ = 5.47 mg/g/h, which was significantly lower compared to the cytoplasmic product. Production of the periplasmic protein started only 6 h post induction and low uptake rates of the inducer lactose potentially explain the low specific productivity during the single-cycle fed-batch. As production of periplasmic products depends on several factors (i.e., translocation to the periplasm), generally lower specific productivity can be expected, compared to protein expression in the cytoplasm (Kleiner-Grote et al., 2018; Karyolaimos et al., 2019). No decrease in viability could be observed throughout cycle one, as for the cytoplasmic product.

Cell specific productivity in the two-cycle repeated fed-batch cultivation was much higher compared to the first cycle (*q*_p_ = 14.44 mg/g/h). Even though viability did not decrease in cycle two, leaky product (7.7% of total product) could be detected in the supernatant. This behavior has been observed for periplasmic proteins in literature before (Chen et al., 2014; Hausjell et al., 2020).

For repeated fed-batch technology carried out for three cycles, a minor decrease in productivity was found compared to cycle two, resulting in a *q*_p_ of 12.96 mg/g/h. The uptake rate of the inducer lactose increased during cycle two and three (Supplementary Figure 4b). Thus, we hypothesize that longer timespans of full lactose induction were the reason for the increase in specific productivity compared to cycle one. No cell death was monitored throughout cycle three, however higher amounts of leakiness were found (28.1% of total product). No fourth cycle was conducted, in order to make repetitive fed-batch cultivations for both target proteins comparable in their number of cycles. Moreover, levels of leakiness increased over the time-span of the cultivation, which was a further reason to terminate the process after cycle three.

For the chemostat cultivation no product formation was found for the first 8 h of induction. Product formation started after 8 h of induction and increased until reaching a peak of 5.75 mg/g/h after 20–25 h. The timespan until full induction was comparable for repetitive fed batch cultivations and chemostat cultivations (Figure 3). Therefore, it seems like the expression of the periplasmic protein required an adaption time after the start of induction, to establish protein translocation toward the periplasm (Kopp et al., 2017). Throughout chemostat cultivation maximum specific productivity was lower compared to repetitive fed-batch cultivation by a factor of 2.9 (which is in accordance with results obtained for the cytoplasmic product). However, we could not monitor any secretion of periplasmic protein during continuous cultivation. Chemostat cultivation was terminated after 90 h of induction as productivity was below the LOD for both products.

Again, a theoretical ideal chemostat was simulated, exhibiting time-independent productivity once cell specific productivity reached q_p, max_ (Figure 3B). Our results favor repetitive fed-batch cultivation mode for the periplasmic target protein over single cycle fed-batch and chemostat cultivations regarding the specific productivity. Achieved space-time yields of each cultivation mode will be compared in section “Targeting Maximum Space-Time Yield: The Cultivation Mode to Choose.”

### Targeting Maximum Space-Time Yield: The Cultivation Mode to Choose

**FIGURE 4:**
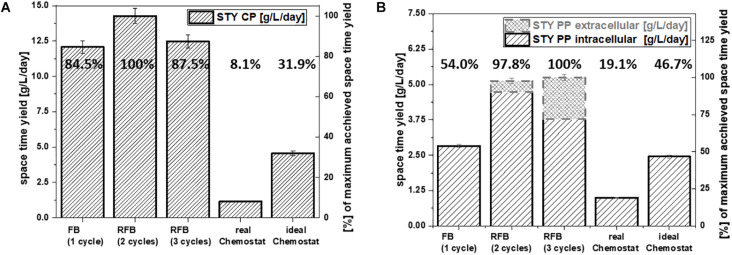
Comparison of the overall space-time yield of each cultivation mode for **(A)** cytoplasmic protein expression and **(B)** periplasmic protein expression in g/L/day; percentages are calculated relatively to the highest space-time yield achieved for each target protein.

The goal of this study was to determine the cultivation strategy giving the highest space-time yield with recombinant *E. coli*. Thus, we calculated the overall space-time yield in g_product_/L_reactor_/day, including “downtimes” for each cultivation mode. Results are shown in Figure 4 and Supplementary Figure 2 for recombinant cytoplasmic and periplasmic protein production.

In fed-batch cultivations, cleaning and set-up take up a severe amount of time. Generally, this leads to a much shorter production time in comparison to the total process time. For repetitive fed-batch cultivations and continuous cultivations these downtimes can be reduced compared to single-cycle fed batches (Figure 1). Even though fed-batch cultivations usually give a high q_p_, cultivation modes with a lower q_p_ in combination with a lower downtime might still result in an increase of the overall space-time yield.

For repetitive fed-batch cultivations and the cytoplasmic target protein, the highest space-time yield was found for a two-cycle process (Figure 4A). Even though q_p_ declined during cycles two and three (Figure 2A), the space-time yield for all repetitive fed batch cultivations was superior to a single-cycle fed batch.

For processes conducted with the periplasmic target protein, q_p_ in cycle two and three was higher than productivity in cycle one (Figure 3A). Hence, it was obvious that repetitive fed-batch would be superior to a single-cycle cultivation regarding space-time yield. Even though the total space-time yield (Figure 4B) differed only by 2.2% when harvesting after cycle two or cycle three for the periplasmic protein, we want to highlight that by applying three induction cycles, total downtime can be reduced compared to the two-cycle repeated fed-batch.

A reduction of downtimes leads to reduced costs for chemicals and energy needed throughout SIP and CIP. Taking into account that CO_2_-taxes for industry will potentially be realized in near future, a reduction of energy consummation could also lead to higher profits (Kettner et al., 2018).

Continuous processes are generally described to lead to higher space-time yields (Lee et al., 2015). However, the monitored space-time yield for the chemostat cultivations within this study was beneath 1/5th of the space-time yield received by the repetitive fed-batch cultivations, independent of the target product (Figures 4A,B). As (i) cell specific productivity and (ii) set biomass concentrations are lower in chemostat cultivation compared to repetitive fed-batch cultivation, this implies an overall reduction of space-time yield for chemostat processes. Product per Substrate Yield at the beginning of the continuous cultivation might compete with repetitive fed-batch cultivations (Tables 4, 5, and Supplementary Table 1). However, a severe decrease in productivity over time was monitored for chemostat cultivations, as microbial chemostat cultivations are known to result in fluctuating productivity (Peebo and Neubauer, 2018; Kopp et al., 2019a).

**TABLE 4:** Comparing substrate per product yield for the production of a cytoplasmic protein; each cycle is investigated separately for fed-batch and repeated fed-batch cultivation; chemostat cultivation is calculated as a rate every 12 h (i.e., one cycle).

induction time (h)	12	24	36	48	60	72	84
cytoplasmic protein	Y_P/S_ (mg/g)						
Fed-batch (1 cycle)	86.8 ± 4.3						
Repeated fed-batch (2 cycles)		84.6 ± 4.2					
Repeated fed-batch (3 cycles)			77.9 ± 3.4				
Chemostat cultivation	241.7 ± 12.1	280.8 ± 14.0	176.3 ± 8.8	11.6 ± 0.6	0	0	0
Theroretical “ideal” chemostat	241.7 ± 12.1	280.8 ± 14.0	280.8 ± 14.0	280.8 ± 14.0	280.8 ± 14.0	280.8 ± 14.0	280.8 ± 14.0

**TABLE 5:** Comparing substrate per product yield in mg/g for the production of a periplasmic protein; each cycle is investigated separately for fed-batch and repeated fed-batch cultivation; chemostat cultivation is calculated as a rate every 12 h (i.e., one cycle).

induction time (h)	12	24	36	48	60	72	84
periplasmic protein	Y_P/S_ (mg/g)						
Fed-batch (1 cycle)	24.5 ± 0.9						
Repeated fed-batch (2 cycles)		135.4 ± 4.8					
Repeated fed-batch (3 cycles)			133.5 ± 4.7				
Chemostat culitvation	94.9 ± 3.3	133.6 ± 4.7	99.3 ± 3.5	58.4 ± 2.1	32.3 ± 1.1	7.8 ± 1.2	5.2 ± 0.8
Theroretical “ideal” chemostat	94.9 ± 3.3	133.6 ± 4.7	133.6 ± 4.7	133.6 ± 4.7	133.6 ± 4.7	133.6 ± 4.7	133.6 ± 4.7

In order to simulate a steady state upon recombinant protein in chemostat cultivation, a stable productivity at q_p, max._ was assumed for more than 6 residence times. The simulated space-time yield however was not superior compared to the repetitive fed batch cultivation. This is because q_p_ of chemostat cultivations was significantly lower compared to repeated fed-batch cultivations (Figures 2, 3). Hence, in this study chemostat cultivation led to a lower productivity and a lower space-time yield and would still need further investigation to achieve the high demands needed for recombinant protein formation.

## Conclusion

The goal of this study was to find out, whether a single-cycle fed-batch, a repetitive fed-batch consisting of two cycles or three cycles or a chemostat is the most suitable cultivation technique to achieve the highest space-time yield of soluble recombinant protein within *E. coli* BL21 (DE3). The impact of the cultivation technology on soluble protein formation was investigated for a cytoplasmic and a periplasmic model protein.

The results of this study show that a repetitive fed-batch approach leads to higher space-time yields compared to single-cycle fed-batches and chemostat cultures. For the cytoplasmic protein a two-cycle repetitive fed-batch was the most efficient cultivation mode, whereas for the periplasmic product a three-cycle repetitive fed-batch was found to be the most efficient cultivation method. Chemostat cultivations suffered from a low maximum specific productivity, which further decreased over time. Therefore, overall product throughput of the chemostat cultivations was much lower compared to other cultivation modes. Furthermore, a single-cycle fed batch was always outperformed by repeated fed-batch independent of the target product and number of applied cycles.

Production processes for recombinant proteins in large-scale are cost-intensive. Here, we were able to show that a repetitive fed-batch cultivation leads to a higher space-time yield compared to a single-cycle fed-batch or a chemostat process. We can promote the developed mixed feeding approach in combination with the repetitive fed-batch cultivation mode, as it leads toward a more economic fingerprint and an increased space-time yield.

## Data Availability Statement

The raw data supporting the conclusions of this article will be made available by the authors, without undue reservation.

## Author Contributions

JK planned the experimental design and carried out the data treatment. SK performed the cultivations and analytics. CS, CH, and OS gave major scientific input. DW founded the idea of this study. JK and DW wrote the manuscript. OS critically reviewed the manuscript. All authors contributed to the article and approved the submitted version.

## Conflict of Interest

The authors declare that the research was conducted in the absence of any commercial or financial relationships that could be construed as a potential conflict of interest.
