# Supplementary material for: Repetitive Fed-Batch: A Promising Process Mode for Biomanufacturing With E. coli
Source: Front Bioeng Biotechnol. 2020 Nov 10;8:573607. doi: 10.3389/fbioe.2020.573607 (PMC7683717; doi:10.3389/fbioe.2020.573607)
Supplement: Supplementary file 1 [file Data_Sheet_1.pdf]

Supplementary Data Section

Repetitive fed-batch: a promising process mode for biomanufacturing with *E. coli*

Julian Kopp[1**], Stefan Kittler[1**], Christoph Slouka[1], Christoph Herwig[1], Oliver Spadiut[1], David J. Wurm[1*]

1. Research Area Biochemical Engineering, Institute of
   Chemical Engineering, TU Wien,
   Gumpendorfer Straße 1a, 1060 Vienna, Austria

*Corresponding author: David J. Wurm

e-mail: david.wurm@tuwien.ac.at

phone: +43-1-58801-166464

[**] these authors contributed equally to the work. JK planned the experimental design and carried out the data treatment. SK performed cultivations and analytics.

Process scheme for cultivation modes applied:

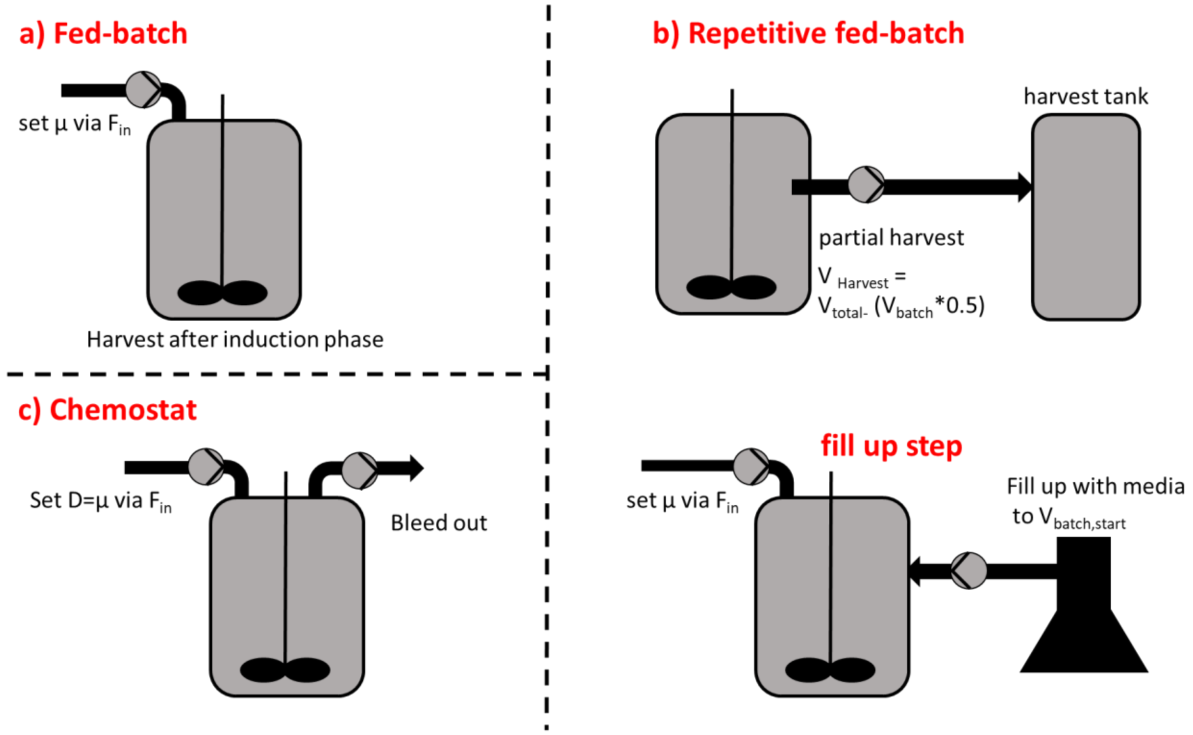

Supplementary Figure 1: Comparison of set ups for cultivation modes of (a) fed-batch, (b) repetitive fed-batch mode including the fill-up step and (c) chemostat cultivation

Time dependent formation of titer throughout repeated fed-batch cultivation:

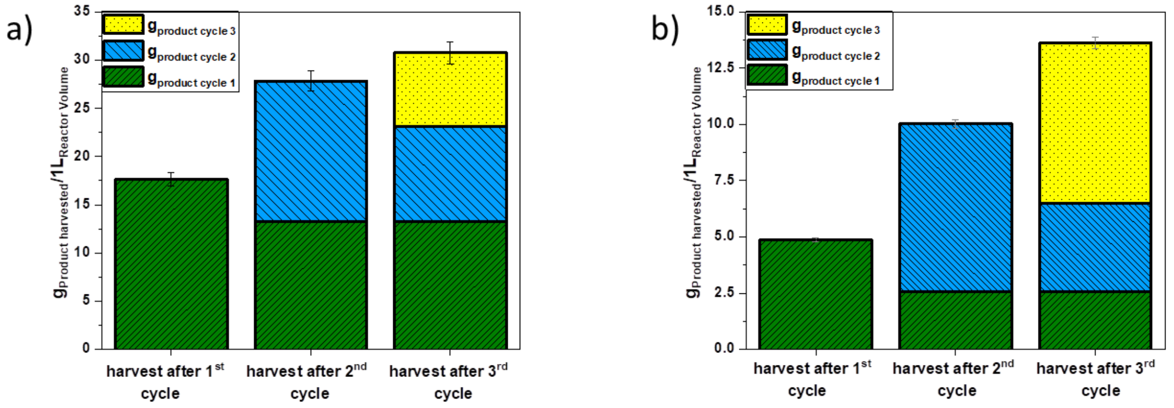

Supplementary Figure 2: showing time dependent formation of titer for repetitive fed-batch cultivations for (a) the expression of a cytoplasmic protein and (b) the expression of a periplasmic protein; As only a partial harvest is conducted different harvests lead to different amounts of product

<u>Time dependent trend of viable cell concentration (VCC) and carbon dioxide evolution rate (CER) of all performed cultivations:</u>

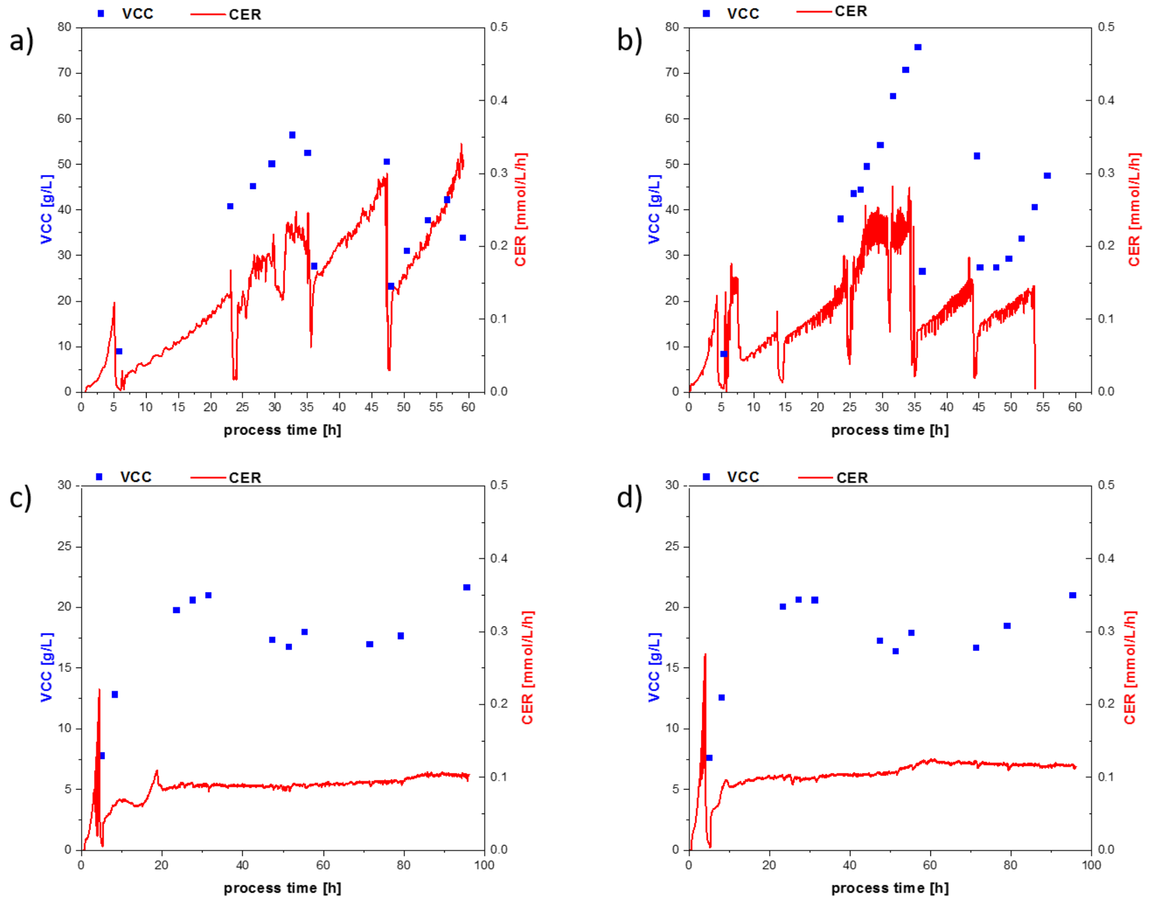

Supplementary Figure 3: showing time dependent trends of viable cell culture (VCC) and the carbon dioxide evolution rate (CER) for repetitive fed-batch cultivations for (a) the expression of a cytoplasmic protein and (b) the expression of a periplasmic protein, and for chemostat cultivations of (c) cytoplasmic protein and (d) periplasmic protein.

<u>Time dependent trend of glucose and lactose of all performed cultivations:</u>

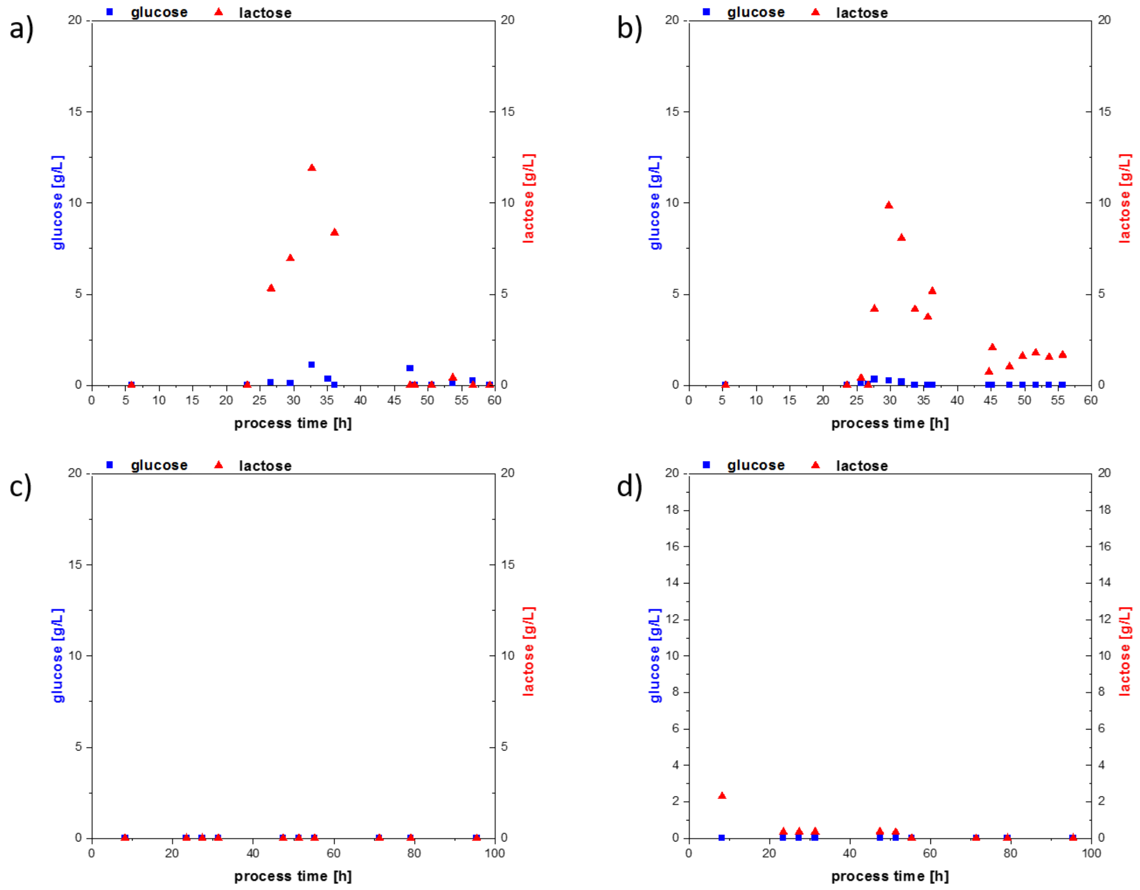

Supplementary Figure 4: showing accumulated sugars in the fermentation broth of the main C-sources (glucose and lactose) for repetitive fed-batch cultivations of (a) a cytoplasmic protein and (b) a periplasmic protein, and for chemostat cultivations of (c) a cytoplasmic protein and (d) a periplasmic protein.

Supplementary table 1: showing a substrate cost estimation for the cost of each fermentation cycle; chemostat cultivation is calculated as a rate every 12 hours (i.e. one cycle); cost estimation was done calculating 0.0064 €/g glucose and 0.0133 €/g lactose working with prices currently given at Carl Roth;

Fermentation cycle	Costs of glucose (€)/ L fermentation broth	Costs of lactose (€)/ L fermentation broth	Total substrate costs (€) / L fermentation broth	Fermentation cycle	Costs of glucose (€)/ L fermentation broth	Costs of lactose (€)/ L fermentation broth	Total substrate costs (€) / L fermentation broth
Fed-batch (1 cycle)	0,82	0,58	1,40	Chemostat 12h induction	0,49	0,40	0,89
Repeated fed-batch (2 cycles)	1,27	1,12	2,40	Chemostat 24h induction	0,87	0,80	1,67
Repeated fed-batch (3 cycles)	1,75	1,68	3,43	Chemostat 36h induction	1,25	1,20	2,45
				Chemostat 48h induction	1,63	1,60	3,23
				Chemostat 60h induction	2,02	2,00	4,02
				Chemostat 72h induction	2,40	2,40	4,80
				Chemostat 84h induction	2,78	2,80	5,58
